# Body Condition Score in Danish Horses Related to Type, Use, and Training Level: Patterns, Risk, and Protective Factors

**DOI:** 10.3390/ani13071219

**Published:** 2023-03-31

**Authors:** Mette Uldahl, Jan Dahl, Hilary Mary Clayton

**Affiliations:** 1 Vejle Equine Practice, Fasanvej 12, 7120 Vejle Øst, Denmark; 2 Jan Dahl Consult, Østrupvej 89, 4350 Ugerløse, Denmark; 3 Sport Horse Science, 3145 Sandhill Road, Mason, MI 48854, USA

**Keywords:** body condition score, equine body condition, training level, horse welfare, body condition score patterns

## Abstract

**Simple Summary:**

The inappropriate body condition of horses is a growing problem that is associated with difficulty both in recognizing and treating horses that are either over-conditioned or under-conditioned. This study is based on visual evaluation by equine professionals and para-professionals (veterinarians, farriers, trainers, Danish Equestrian Federation officials). Participants were trained to assign a body condition score (range 1–9) based on the Henneke Body Condition Score (BCS) system. After watching an instructional video and the satisfactory completion of a questionnaire, they became data collectors. The results indicated that out of 1118 horses evaluated, 78.6% were within, 4.9% were below, and 16.5% were above the ideal range of BCS. Being above the ideal range was influenced by type, age, and training, but none of the patterns influenced the horses below ideal BCS. The highest scores were awarded by veterinarians and farriers and the lowest scores by DEF officials. This was thought to reflect the equine populations they were exposed to, with DEF officials examining actively competing horses whereas veterinarians and farriers see horses with diseases, such as laminitis, that tend to be associated with high BCS. Cold-blooded horses and traditional ponies were more frequently above ideal BCS compared to other types.

**Abstract:**

Body condition in horses is a growing concern that has different patterns of development in horses that are above and below the ideal range. This study used professional and para-professional evaluators (veterinarians, farriers, trainers, Danish Equestrian Federation (DEF) officials) who were trained and certified in the use of a modified Henneke scoring system to assign a body condition score (BCS) on a scale of 1–9. Scores of 5–6 are regarded as ideal, and 78.6% of the evaluated horses were in these groups. Only 4.8% of horses were below ideal BCS but 16.5% were above ideal BCS, and this was influenced by type, age, and training. A significant protective effect towards above ideal BCS was shown for horses trained at higher intensities. Cold-blooded horses and traditional ponies had increased risk for being above ideal BCS. Although BCS increased with age, a large proportion of geriatric horses were both above and below ideal BCS. Discipline was not related to BCS. Patterns of BCS distribution for horses attended by different professionals were investigated. Veterinarians attended more horses with BCS above and below ideal values, farriers mostly saw horses that were above ideal BCS, and officials at competitions mainly saw horses with ideal BCS.

## 1. Introduction

The management of body weight in horses poses a problem for both horse owners and professionals advising on strategies for weight control. Obesity in general, and particularly in certain breeds of horses, poses a significant problem [1,2,3,4].

Obesity is linked to a range of diseases and conditions, including insulin resistance, equine metabolic syndrome (EMS), and laminitis, which potentially affect the welfare and life span of a horse [1,5,6]. The evaluation of the generalized body condition score (BCS) and localized BCS (i.e., neck crest score) is beneficial in predicting laminitic episodes [7]. Reproductive efficiency also depends on the ratio of body fat to non-fat components [8,9,10]. In horses, the use of weight tracking systems, by the owner, has a protective effect against euthanasia [5]. Further, exercise has been shown to help improve insulin sensitivity, which contributes to a healthy weight [11].

Consideration has been given as to whether modern methods of managing leisure horse have an inbuilt tendency to promote obesity as part of a multiple factor relationship, where diet, use (companionship rather than competition), and exercise are contributory factors [12,13,14]. Horse owners often have difficulty evaluating the body condition of their horses; they may misinterpret a fat body shape as being normal or confuse fat with muscles [2]. A further problem is that owners have difficulty managing obesity in horses [2], and weight gain can occur unintentionally, even when owners are aiming for maintenance or loss [15]. There is a tendency to consider the horse’s ideal weight to be affected by the type of work or the sporting discipline the horse is used for, with over-weight being perceived as ideal (normal) for some disciplines, e.g., showing [16]. There is a relationship between the workload and the likelihood of being over-weight; pleasure horses are less likely to become over-weight than horses not used for any type of work. Competition horses, or horses in intense training, have an even lower risk of obesity [17].

Apart from the negative health and welfare consequences, the average costs for owners of obese horses are higher than those for horses of an ideal weight due to additional expenditures for veterinary care, special feed, extra costs for fencing, and other actions taken to deal with the over-conditioning or obesity problem [18]. Further, horses experiencing laminitis due to over-weight and/or other factors require intensive and often complex management [18], including frequent trimming and shoeing procedures by experienced farriers [19]. For example, the risk for laminitis has been shown to more than double with weight gain [15].

A study of 254 Icelandic horses in Denmark found that 24% of the horses exceeded normal BCS: 13.8% were classified as over-weight (BCS 7), and 10.2% were obese (BCS 8–9) [20]. By comparison, only 5.9% of the horses were under-weight (BCS 3–4) [20]. A Swedish analysis of official animal welfare control data showed that both obese and under-weight horses presented as a welfare problem in official controls [21]. Both studies illustrate that an overall perspective on horse weight needs to consider horses that are both below and above the ideal weight. Indeed, a study of factors associated with the mortality of geriatric horses in the United Kingdom considered those that were under-weight to have a greater risk of mortality than those in good condition [22].

Age influences weight, with horses older than 4 years being at a greater risk of becoming over-weight [23]. This is probably due to young horses growing and being more active than older horses. Historically, senior horses were prone to be under-weight due to geriatric problems, such as dental issues, exercise intolerance, and metabolic dysfunction [24], but with modern management, there are fewer problems with under-weight senior horses. It is important to recognize normal BCS in different populations in order to identify structural patterns of non-ideal BCS in horses, as, for example, the risk for laminitis has been shown to more than double with weight gain [7].

The gold standard for the assessment of body fat includes the direct objective evaluation of adipose tissue. This can be performed by dissecting carcasses [25,26] or by ultrasonography of subcutaneous fat [27,28]. Objective measurements of body size, such as morphometric measures [29], are an alternative objective standard. However, in many settings, refined objective measures are not practical in daily life for individual horse owners or if a large number of horses are to be evaluated. For practicality, and to broaden the scope for use, subjective methods for the assessment of body condition and body fat accumulation in horses have been developed.

The Henneke body condition scoring system is commonly used for this purpose. It is based on a 9-point (1–9) system [16,17] in which particular anatomical areas are selected as being indicative of changes in stored body fat, including the neck, withers/area behind the shoulder, ribs, spinous processes, and lumbar and tail regions. These areas are inspected visually, palpated, and then given a score from 1 (extremely emaciated) to 9 (extremely obese) [30,31]. A score of 5 is moderate (ideal) [31]. Scores lower than 5 indicate that horses are progressively thinner (below ideal). A score of 6 is moderately fleshy [31], and scores from 6 to 9 are awarded to horses that are fatter/obese [31] (above ideal). Scores of 5 and 6 are within the optimal range of BCS [20].

The validity of one of the subjective body condition scoring methods that is based on a 0–5 scale has been evaluated [32]. The results showed a good correlation between BCS values awarded by owners and an experienced veterinarian. In this study, the owners were not taught how to use the scoring system but were given graphical illustrations to aid in score assignment [32].

The objective of this study was to determine the distribution of body condition scores across subpopulations of a large number of horses in Denmark, including the prevalence of scores below and above ideal BCS. Further, the parameters sex, age, breed/type, use, and level of training were included to assess their effects on relative risk or as a protective factor.

## 2. Material & Methods

### 2.1. Study Design

The body condition scores of 1118 horses and ponies were evaluated between June 2021 and November 2021. Most of the data were collected at Danish Equestrian Federation (DEF) events (770 horses), and the remainder of the data were collected from horse yards (348 horses).

Professional and semi-professional data collectors (24) were recruited by the main author (MU), comprising veterinarians (5), feed consultants (5), farriers (2), equine therapists (2), trainers (3), and Danish Equestrian Federation (DEF) officials (7).

People who applied to be registered as data collectors were sent a link to an instructional video and to a mock online questionnaire. It was mandatory to watch the instructional video at least once before collecting data for the survey. Applicants performed at least two mock examinations of horses, including online questionnaires, before returning a confirmation by mail that the content of the instructional video and the concept of filling out the questionnaires were understood. The answers on the submitted mock questionnaires were checked by the main author (MU) and, if the answers were satisfactory, a link to the study’s online questionnaire was forwarded along with formal acceptance as a data collector.

The 3-min mandatory instructional video informed the data collectors how to fill out the online questionnaire for each individual horse, including a general consent statement for the collection of data for research that was read aloud to the horse owner or person responsible for the horse. Acceptance of the consent statement was mandatory before the visual examination of a horse during collection in horse yards. For officials collecting data at DEF event sites, the video explained how to announce and secure the obligatory consent for data collection according to DEF rules. Further, the importance of recording the individual horse identification numbers from their microchip, passport or by online accessing of their license at the DEF competition website was explained together with all other parameters to be recorded.

The assignment of scores ranging from 1 to 9 according to the Henneke body condition scoring system was explained by displaying focus areas for the assessment of fat deposits in the photos of the horses. Examples were shown to illustrate the range of the scale. The importance of looking at the horse from a distance and closer up and the evaluation of fat deposits in the neck, shoulder, withers, ribcage, hind quarters, and tail region were explained. Tips for assessment were highlighted:

- If the ribs are clearly protruding, the category is <5;
- If the ribs are not visible, the score is ≥5;
- Fat in the neck or shoulder region is often seen with BCS >6;
- If the vertebrae and pelvic bones are visible, the score is ≤4;
- If the vertebral spine is level with the musculature, the score is 5–6;
- If the vertebrae are recessed into a hollow between fat deposits on either side of the midline, the score is 7–9.

**Figure 1:**
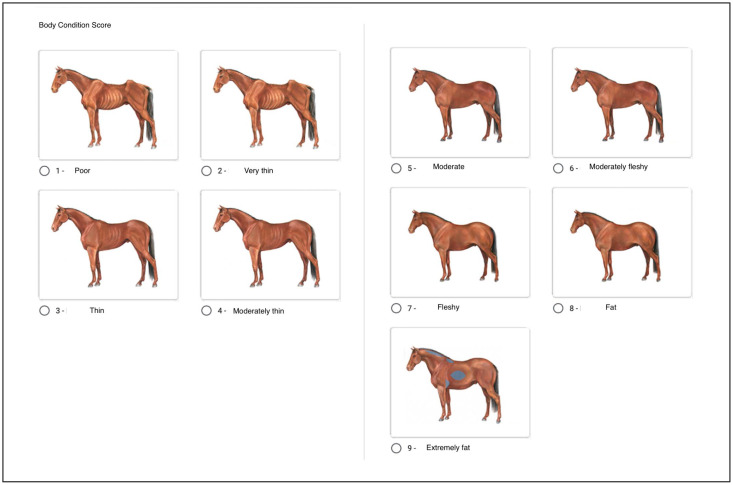
Illustration of the appearance of horses for each body condition score, from the Henneke scale 1–9.

As conclusive remarks, the data collectors were instructed to make an overall assessment of the horse after having inspected each highlighted area visually (see Appendix A and Appendix B). A graphic illustration scheme of horses (from scale 1–9) with descriptive text for each category was included in each questionnaire as a tool for assessment (see Figure 1).

The following data were collected in a survey format using Google Analysis (data collectors filled out online questionnaires):

General information of how to watch the instruction video and fill out the questionnaire.

Instructional video (link to a YouTube presentation at the beginning of each on-line questionnaire).

Identity of the data collector.

Type of consent/Recording of consent from the horse owner/Site of collection (including the name of the project administrator, Mette Uldahl, and a standard statement text to be read aloud followed by tick boxes with two options: type “1” for oral consent from a private horse owner when data were collected from horse yards, type “2” for consent by DEF and the event organizer with an announcement made prior to the collection of data and according to DEF rules at competitions).

Horse Identity (Unique Equine Life Number or microchip number).

Sex (Mare, gelding, stallion).

Year of birth.

Type of horse: sport horse, traditional horse, cold-blooded horse, Baroque horse (Friesian, Lippizan, Iberian breeds), sport pony, traditional pony, small horses and mountain breeds, Icelandic horses, trotters, thoroughbred race horses, western horses.

Discipline/intended use (show jumping, dressage, eventing, endurance, competition Icelandic, trotter racing, thoroughbred racing, leisure, breeding, showing, school pony/horse, no discipline, other). More than one discipline/intended use can be recorded.

Training intensity in daily work (no training, light, moderate, high, very high; see Table 1).

Body condition score (Henneke scale 1–9; tick the box with a graphical illustration of BCS similar to the horse).

After data collection, different categories for the two parameters “type of horse” and “discipline” were merged due to there being only a few observations:

Merged groups for “Type of horse”: Riding horses (Horse sport (Warmblood, Trakehner, Danish Palomino Sport horse, Arabian, Pinto), traditional/old style horse (Oldenburg, Knabstrupper, Frederiksborger), Baroque (Friesian/Lippizan/Iberian), western (Paint, Quarter horse)); sport pony (Sport pony, Connemara, New Forest, Welsh); traditional pony (Dartmoor, Gotland Russ, Shetland etc.).; Cold-blooded (cold blood/draft style (Jysk, Belgian, Shire, Tinker, North Swedish), small robust horses (Norwegian Fjord Horse, Haflinger)); Icelandic; Racing (Trotter, thoroughbred).

Merged groups for “Discipline/intended use”: Showjumping; Dressage; Mixed disciplines: *eventing, endurance, mixed/combined riding disciplines*; Icelandic competition; Hacking: *hacking/leisure, other*; Breeding; Riding school horse/pony; No discipline. The groups trotting, racing, and showing were deleted due to no recordings of horses being active in those disciplines/intended use.

### 2.2. Statistical Analysis

Initially, a bivariate analysis of BCS and predictors was performed. As some of the data groups had few observations, it was decided, prior to analysis, to merge compatible groups to increase the statistical power. A graphical representation of the results was made using proc univariate (SAS Inst.). The statistical significance of the bivariate analysis was performed using proc npar1way (SAS Inst.). An expected clear non-normality of the scores was observed, and based on this finding, the Kruskal–Wallis test was used to test for significance.

The data were analyzed with a multinominal logistic regression model with ordered data (ordinal scale), using the cumulative logit link (proc genmod, SAS Inst.). The training level, profession of the data collector, and age were continuous variables. The type of horse, discipline/intended use, sex, and site of collection were fixed effects. Data collector was included as a repeated factor due to an assumption that differences between collectors could be expected.

After an initial multinominal logistic regression analysis, “profession of data collector” was excluded, as it showed a very high level of correlation with “site of collection/type of consent”. The remaining factors were retained in the model even if they were not significant due to confounding effects between factors.

An additional analysis was made where BCS categories were merged into three groups: ideal (BCS 5,6), above ideal (BCS > 6), and below ideal (BCS < 5). The rationale behind this was that the ordinal scale of BCS may not reflect the same mechanisms in horses scoring below the ideal BCS vs. horses scoring above the ideal BCS. Mechanisms that increase the risk of being below ideal are likely different from the mechanisms that decrease the risk of being above ideal.

At first, simple contingency tables were made with *p*-values based on chi-square tests (Fisher’s exact test was used if there were fewer than five observations in a cell). A multinomial logistic regression model (proc glimmix, SAS Institute) was performed, with ideal BCS as the reference value, and below and above ideal BCS as categories. The modelling approach followed the same procedure as the ordinal analyses. Data collector was introduced as a random variable.

For explanatory variables with more than two levels and a *p*-value below 0.10 for the overall effect of the variable, further analyses were performed to investigate if there were groups that differed significantly from the rest of the groups. This was done stepwise, so the group with the largest difference from the average BCS was tested against the remaining groups. If this was significant, the procedure was repeated, comparing the next group to the remaining groups. This procedure continued until the *p*-value was non-significant.

## 3. Results

### 3.1. Distribution of BCS in the Population

The distribution of the BCS data in total was as follows: 1 (0.0%), 2 (0.0%), 3 (0.5%), 4 (4.4%), 5 (53.6%), 6 (25.0%), 7 (8.8%), 8 (4.6%), and 9 (3.1%).

For the overall population, no horses were cachectic (1–2), 4.4% were below ideal BCS (3–4), 78.7% had ideal BCS (5–6), and 16.5% were above ideal. The number of horses above ideal (184) was 3.4 times more than the number below ideal (54).

### 3.2. Data Collectors and Site of Collection/Type of Consent

Overall, 24 data collectors collected data for the study. On average, they collected 46.6 questionnaires each (range 2–169). The breakdown of data collectors by profession and the number of horses recorded by each group were as follows: five veterinarians (88), five feed consultants (118), two farriers (91), two horse therapists (25), three trainers (26), and seven DEF officials (770).

**Figure 2:**
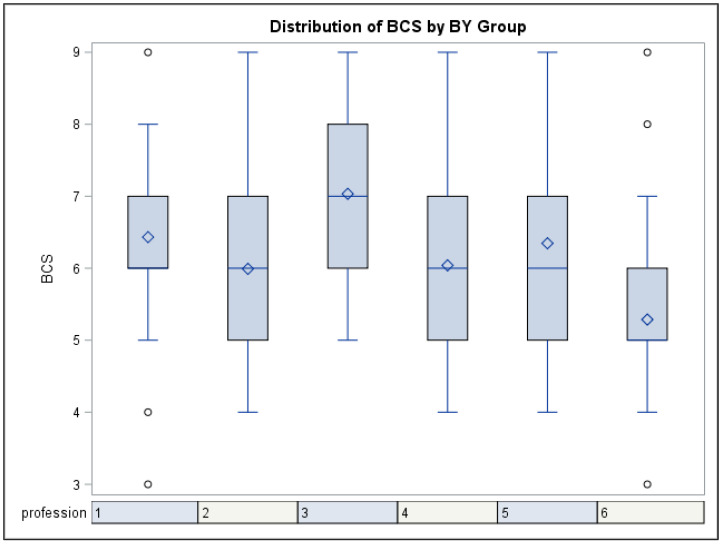
Kruskal–Wallis plot, distribution of BCS for data collectors’ profession. 1: Veterinarians, 2: Feed consultants, 3: Farriers, 4: Equine therapists, 5: Trainers, 6: DEF officials (*p* = 0.001). The box represents 50% of the scores for the group. The blue line in the box is the median, and the diamond is the mean. The upper and lower blue lines show the minimum and maximum values. Circles outside the lines represent outliers.

Farriers recorded the highest average BCS of 7.0 (range 4–9), followed by veterinarians with an average of 6.4 (range 5–8), trainers with an average of 6.3 (range 4–9), and feed consultants and horse therapists with an average of 6.0 (range 4–9). DEF officials had the lowest average BCS of 5.3 (range 4–7) (Figure 2).

**Figure 3:**
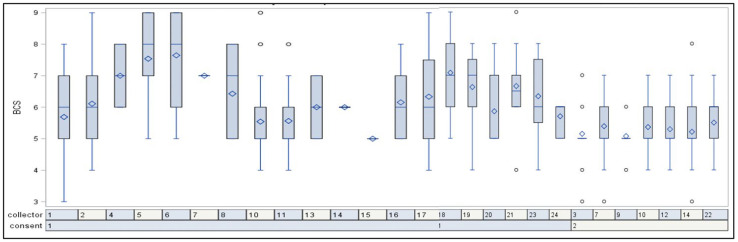
Distribution of the body condition score per collector. The distribution for consent type 2 is evenly distributed (DEF officials scoring horses at events). The box represents 50% of the scores for the group. The blue line in the box is the median, and the diamond is the mean. The upper and lower blue lines indicate the minimum and maximum values. Circles outside the lines represent outliers.

A random effect of individual data collectors was found after adjustment for profession (*p* < 0.0001). When presenting BCS per collector, it was shown that the profession “DEF officials” was evenly distributed in comparison to individual collectors from other professions; see Figure 3.

Further analysis of the categories “ideal BCS”, “above ideal”, and “below ideal” showed that farriers scored more horses above ideal (61.5%) compared with veterinarians, trainers, and therapists (46.6%, 46.2%, 36.0%), with the latter group also scoring the largest proportion of horses below ideal BCS (9.1%, 7.8%, 12.0%). Officials and feed consultants mostly scored horses as having ideal BCS (90.4% and 72.0%), although feed consultants also scored a proportion of horses as above ideal (26.3%).

However, after adjustment for the site of collection (type 1: horse yards; type 2: competition), the effect of profession was not significant (*p* = 0.69) (Table 2). Since only DEF officials recorded scores for horses in competition, there was a relationship between the site of collection and type of consent.

At competitions, 90.7% of horses had ideal BCS, with 4.0% above ideal and 5.4% below ideal compared to 57.8% of horses with ideal BCS in horse yards where 38.1% were above ideal and 3.9% were below.

Horses in horse yards (average 6.3, range 3.9) scored on average higher than horses at competitions (average 5.3, range 4–7). The odds for scoring higher in the multivariable analyses, ordinal scale, for horses in horse yards was 3.26 (c.l. 1.71–6.23). On the nominal scale, the odds for scoring above ideal BCS were 7.69 (c.l. 1.62–33.33) times higher in horse yards. There was no significant difference for the odds of being below ideal (Table 3).

OR based on the multivariate multivariable analysis on an ordinal scale showed a significantly higher risk of increased BCS for horses in horse yards than in competition, OR = 3.3 (*p* = 0.003) (Table 3).

OR based on the additional multivariable analysis on a nominal scale showed a significantly decreased risk of above ideal BCS in competition, OR = 0.13 (*p* = 0.01), relative to horses in horse yards (Table 4).

OR based on the multivariate multivariable analysis, nominal scale, did not show a significantly increased risk of being below ideal BCS in competition relative to horse yards (*p* = 0.31) (Table 4).

### 3.3. Sex

The study included 92 stallions, 582 geldings, and 444 mares. The above ideal category (BCS > 6) included 6.5% of stallions, 16.3% of geldings, and 18.7% of mares, while the below ideal category (BCS < 5) included 6.5% of stallions, 5.0% of geldings, and 4.3% of mares.

Sex was significantly related to BCS in a Kruskal–Wallis bivariate analysis, ordinal scale (*p* < 0.0188), and trending in nominal scale (*p* = 0.0696). Sex was not significantly related to BCS in the multivariable analysis for either the ordinal scale or the additional multivariable analysis, nominal scale (*p* > 0.05) (Table 2 and Table 5).

### 3.4. Type of Horse

The distribution of horses in the study was 542 sport horses, 52 traditional/old style horses, 8 cold-blooded, 13 Baroque/Friesian/Lipizzaner, 300 Sports ponies, 28 traditional/old style ponies, 21 small/robust, 146 Icelandic, 2 trotters, 5 thoroughbreds, and 1 western paint.

In the merged groups, used for the bivariate and multivariable analyses, the distribution was 29 cold-blooded types, 146 Icelandic, 7 race horses, 300 sports ponies, 28 traditional/old style ponies, and 608 riding horse types.

The type of horse was significantly related to BCS in a Kruskal–Wallis bivariate analysis, ordinal scale (*p* < 0.0001), and the chi-square test for the nominal scale (*p* < 0.0001). The type of horse showed a trend in the multivariable analysis, ordinal scale (*p* = 0.0643), and was close to significance in the multivariable analysis, nominal scale, (*p* = 0.0504) (Table 2 and Table 5).

In the additional analysis of the categories ideal, above ideal, and below ideal BCS, the contingency table showed the highest proportion of ideal BCS for sport ponies (83.0%) and riding horses (84.7%). Race horses had a relatively high percentage of below ideal BCS (28.6%), but this was based on low numbers. The highest proportion of horses with above ideal BCS was recorded for cold-blooded types (79.3%) and traditional ponies (46.4%) (Table 6).

OR based on the multivariable analysis, ordinal scale, showed a significantly increased risk of higher BCS for cold-blooded horses compared to the non-cold-blooded horses, OR = 12.7 (C.I. 3.43–46.91). Traditional ponies also had a significantly increased risk compared to non-traditional ponies, OR = 2.95 (C.I. 1.18–7.38) (Table 3).

### 3.5. Age of Horse

The distribution of horses was by age was 135 horses <5 years old, 460 horses 6–10 years old, 326 horses 11–15 years old, 130 horses 16–20 years old, 56 horses 21–25 years old, and 11 horses 26–30 years old.

Age was significantly related to BCS in the Kruskal–Wallis bivariate analysis, ordinal scale (*p* < 0.0001), and in a chi-square test, nominal scale (*p* < 0.0001). Horses younger than 5 years had the lowest BCS value with an average of 5.4 (range 4–7) followed by 6–10 year-old horses, average 5.5 (range 4–7), 11–15 year-old horses, average 5.8 (range 4–7), 16–20 year-old horses, average 5.9 (range 3–9), and 21–25 year-old horses, average 6.1 (range 4–9). The age group 25–30 years showed a decline in average BCS relative to those aged 21–25 years, with an average of 5.7 (range 3–8).

Age was also significantly related to BCS in the multivariable analysis, ordinal scale (*p* = 0.0030), with a trend in the additional multivariable analysis, nominal scale (*p* = 0.0858). In the additional analysis of the three BCS categories, the percentages shown in the contingency table indicated that above ideal BCS increased with age: ≤5 years old = 9.6%, 6–10 years old = 11.3%, 11–15 years old = 19.0%, 16–20 years old = 26.2%, 21–25 years old = 33.9%, and 26–30 years old = 36.4%. Below ideal BCS was stable between age groups: <5 years old = 5.2%, 6–10 years old = 3.3%, 11–15 years old =6.1%, 16–20 years old = 3.9%, 21–25 years old = 7.1%, apart from an increase for the group of 26–30 years old = 27.3% (*p* = 0.0001) (Table 7).

OR based on the multivariable analysis, ordinal scale, showed a significantly increased risk of higher BCS for horses per additional year, OR = 1.06 (*p* < 0.0001) (Table 3).

OR based on the multivariable analysis, nominal scale, showed a trend of an increased risk of being above ideal per additional year, OR = 1.05 (*p* = 0.06) (Table 4).

### 3.6. Discipline/Intended Use

Discipline was significantly related to BCS in the Kruskal–Wallis bivariate analysis, ordinal scale (*p* < 0.0001), and the chi-square test for the nominal scale (*p* < 0.0001). Riding school horses had the relatively highest BCS of the categories with mean 6.7 (range 5–8) followed by hacking (mean: 6.5; range: 4–9), Icelandic horse (mean: 6.4; range: 4–9), no discipline (mean: 6.2; range 3–9), mix (mean: 5.9; range: 5–7), breeding (mean: 5.8; range 4–7), dressage (mean: 5.4; range 4–7), and show jumping (mean: 5.2; range 4–7).

Discipline was not significantly related to BCS in the multivariable analysis, nominal scale (*p* = 0.3093), or the additional multivariable analysis, ordinal scale (*p* = 0.4938) (Table 5).

### 3.7. Training Level

Training level was significantly related to BCS in the bivariate analysis, ordinal scale (Kruskal–Wallis *p* < 0.0001); see Table 2.

Horses with no training (level 1) had the relative highest BCS with an average of 6.3 BCS (range 3–9), followed by light training (level 2), average 6.4 (range 4–9); moderate training (level 3), average 5.4 (range 3–7); a high level of training (level 4), average 5.3 (range 4–9); and a very high level of training, level 5, average 4.8 (range 3–6).

The training level was significantly related to BCS in both the multivariable analysis, ordinal scale (*p* = 0.0093), and in the additional multivariable analysis, nominal scale (*p* = 0.0460). See Table 5.

In the additional analysis of the categories ideal, above ideal and below ideal, the percentages shown in a contingency table indicated that the proportion of horses with ideal BCS increased with higher levels of exercise; the proportion of above ideal BCS increased with lower levels, whereas that below ideal was consistent among the groups apart from an increase at very high levels of exercise (however, only a few horses were included in the group); see Table 8.

OR based on the multivariable analysis, ordinal scale, showed a significant decrease in BCS for horses when increasing +1 level of training on the scale of 1–5, OR = 0.66 (*p* < 0.0001) (Table 3).

No significant changes were identified in relation to BCS and training for below ideal BCS horses, OR 0.94 (*p* = 0.87) (Table 4).

## 4. Discussion

Weight management in horses is a growing concern [1,2,3,4] due to the increasing number of horses suffering from conditions related to being over-weight or obese [1,5,6,8,9,10]. This study investigated the body condition score related to horse type, use, age, and training level. We found that 4.8% of horses had below ideal BCS whereas horses with above ideal BCS had a higher prevalence of 16.5%, and this was influenced by type, age, and training. There were 3.4 times as many horses with above ideal BCS than those with below ideal BCS.

In a study from 2016 that was based on visual assessment and palpation, 24% of Icelandic horses in Denmark were above the ideal BCS [20]. Our study, based only on visual assessment, found that 33.6% of Icelandic horses were above the ideal weight, suggesting that the problem is worsening. Although equine obesity is currently a topical issue, horses with BCS below the ideal value are also a welfare problem for the individual animal, and the underlying pattern and risk factors should be investigated.

### 4.1. Data Collectors: Patterns within Professions

In the present study, data collected by professionals and para-professionals within the equine industry were used to investigate patterns within and across professions. Prior to collection of data, all data collectors were certified for competency in the techniques to ensure an even level of data quality but, even so, there was variability in scores even though these were not significant when adjusted for the site of collection. Further, a random effect of individual data collectors was found after adjustment for profession.

The variation in scoring between professions is most likely caused by the variation in the distribution of BCS within the subpopulations attended by the individual professions, as the effect of profession became statistically non-significant after control for collection site. On average, farriers and veterinarians recorded higher BCS than the other professionals, and DEF officials at competitions scored the lowest. DEF officials exclusively evaluated sport horses and ponies in competition where horses in the ideal range of BCS constituted 90.4%. Trainers saw horses of a wider spectrum of condition (46.2% above ideal, 46.2% ideal, and 7.7% below ideal). Veterinarians and therapists regularly attend horses with health problems, where risk factors are influenced by the horse being either over-weight or under-weight. In this study, they scored a relatively high proportion of above ideal BCS (46.6% and 36.0%) and also below ideal (9.1% and 12.0%). Farriers attend horses with hoof problems, such as laminitis, more frequently than horses with healthy hooves [19]. Laminitis is related to over-weight horses [5,18]; in the present study, above ideal BCS constituted of 61.5% of recordings from farriers.

Interobserver variability between data collectors can be evaluated by looking at the DEF officials’ scores for the group of horses expected to be the most evenly distributed (competition horses). It was seen from a Kruskal–Wallis plot that the profession “DEF officials” had an even distribution of recordings between observers.

### 4.2. Body Condition Score Recorded for Horses in Competition vs. Those in Horse Yards

The modern management of leisure horses potentially has an inbuilt incentive towards obesity, made up of a multiple factor relationship where diet, use (companionship rather than competition), and exercise are significant factors [12,13,14]. This hypothesis is supported by findings in this study, where patterns were identified for horses assessed in competition compared to those evaluated in horse yards. On average, a high proportion of horses in competition had ideal BCS (90.7%) compared to those assessed out of competition (57.8%). Above ideal BCS was recorded in only 4.0% of horses in competition relative to 38.1% in horse yards.

A relationship between a horse’s regular workload and the likelihood of being over-weight has also been shown in previous studies; pleasure horses are less likely to become over-weight than horses not used for any type of work, and competition horses, or horses in intense exercise routines, have an even lower risk of obesity [17]. The protective effect against above ideal BCS and being a competition horse is most likely due to the coupled effects of use for competition and training for competition derived from the intended use. In the present study, horses in horse yards had 3.26 higher odds of having higher BCS compared to horses in competition. A relative decrease in the risk for having above ideal BCS was shown for horses in competition, OR = 0.13.

The difference between the number of horses having below ideal BCS was less pronounced; 5.4% in competition and 3.9% in horse yards.

### 4.3. Distribution of BCS, including Patterns for Ideal, below Ideal, and above Ideal BCS

There is a relationship between negative health and welfare consequences and increased average costs for owners of obese horses. Additionally, studies have shown higher costs for professionals and products, such as veterinary care in horses with above ideal BCS [18]. To avoid these additional costs and complications, it is important to identify the structural patterns of non-ideal BCS in horses that have been linked to animal welfare problems [21]. For example, the risk for laminitis is more than doubled with weight gain [15], and above ideal BCS was recorded 3.4 times more often than below ideal BCS.

The overall distribution of BCS showed no recordings of cachectic horses (BCS < 3), and only 54 of 1118 horses (4.8%) were below ideal BCS (3–4). This compares with 880 horses (78.7%) that were within the ideal range (5–6) and 184 horses (16.5%) that were above ideal.

#### 4.3.1. Above Ideal BCS

Owners frequently find it very difficult to manage obesity in horses [2], and weight gain often occurs unintentionally, even when owners are aiming for maintenance or weight loss [15]. This is consistent with the observation that weight has been shown to increase with age in both this and previous studies [23]. In this study, an increased risk per additional year of 1.06 (0–30 years old) was found, with 9.6% of the 0–5 year olds having above ideal BCS, increasing to 36.4% in the 26–30 year olds.

Riding school horses had the highest relative BCS, with an average of 6.7, followed by hacking, Icelandic competition horses, no discipline, mixed disciplines, and breeding. Dressage (5.4) and show jumping (5.2) had the lowest ranking in BCS on average. However, the effect of discipline was highly correlated with the type of horse and was not significant. Cold-blooded horses and traditional ponies had an increased risk of higher BCS compared to the other groups.

In the present study, training level was a significant factor in relation to BCS. Horses, not being trained or only undergoing light training, had the highest relative BCS, followed by horses in moderate training, while high and very high training levels resulted in the lowest BCS. Increased training level reduced the odds for having higher BCS. Going from low to moderate exercise levels, the proportion of horses with above ideal BCS decreased from 44.0% to <10%. Exercise is equally shown in other studies to help improve insulin sensitivity, which contributes to a healthy weight [11].

Sex was not significantly related to BCS.

#### 4.3.2. Below Ideal BCS

The pattern for below ideal BCS was different than for above ideal BCS, which is important to recognize, as under-weight is linked to a greater risk of euthanasia, for example, in geriatric horses [22], but with different associated risk factors than for over-weight.

In this study, below ideal BCS was relatively stable between age groups, ranging from 3.3% to 7.2%, apart from an increase to 27.3% for the group of 26–30 year-old horses. Historically, senior horses were prone to be under-weight due to geriatric problems, such as dental issues, exercise intolerance, and metabolic dysfunction [24]. With modern management, fewer problems with under-weight senior horses are seen, but it seems there is still potential for improvement.

The odds for below ideal BCS increased per year of age, but the effect was not significant (OR = 1.06, *p* = 0.21). For horses below ideal BCS, no correlation was shown for recordings at competition versus horse yards. Further, neither discipline nor type of horse was significantly correlated. Training did not significantly influence horses below ideal BCS positively or negatively (OR = 0.94). Sex was not significantly related to BCS; numerically, it was shown that 6.5% of stallions, 5% of geldings, and 4.3% of mares were below ideal (BCS < 5).

The prevalence of below ideal BCS corresponds well with findings in a comparable study [20]. Below ideal BCS displayed a completely different pattern than above ideal BCS.

### 4.4. Direct Objective Evaluation of Body Fat Storage vs. the Use of Subjective Scales for Body Condition Score: Strengths and Limitations

The gold standard for the assessment of body fat includes a direct objective evaluation of adipose tissue in carcasses [25,26]. In living horses, methods such as ultrasonography of subcutaneous fat are used [27,28]. Morphometric measures of body size are an alternative objective standard [29]. However, in many settings, refined objective measures are not practical for use by individual horse owners or if a large number of horses are evaluated. Therefore, different subjective methods have been developed for the assessment of body condition and body fat accumulation in horses where the horse’s body condition score is evaluated.

A subjective assessment of individual BCS values for horses can be performed both visually and by palpation of specific regions of the horse’s body. Both localized (i.e., neck crest score) and general scoring of the body condition can be used as a preventive measure and can help predict laminitic episodes [7]. Further, a protective effect towards euthanasia has been proven [5]. When using BCS as a potential health measure for horses, uniformity in the individual scorings is essential. A study of the validity of subjective body condition scoring methods reported a good correlation in equine BCS data between owners and an experienced veterinarian [32].

The Henneke body condition score system is a commonly used 9-point system with a range from 1 to 9 [30,31]. In this study, visual assessments based on the Henneke scale were made. It is known that it can be difficult for horse owners to evaluate their horse body condition, as they often misinterpret a fat body shape for a regular shape of the horse, or confuse fat with muscles [2]. In this study, the assigned data collectors were professionals and para-professionals within the horse industry who had been trained to use a systematic protocol before the collection of data. This included an assessment of the overall body conformation with a specific assessment of key regions to ensure an even methodology for scorings across the population. Further, the graphic illustrations used in the study provided a visual reference to help select the correct score for each individual horse. However, it can be a limitation that visual scoring was exclusively used in the study compared to a combination of visual evaluation and palpation.

With regards to the mapping of patterns and risk factors overall, another limitation of the study is the lack of recording of the horse’s feed. This parameter was excluded because it did not fall within the primary objective of the study, which was to measure the effects of intended use and training level. The recording of individual feeding regimes and quantities would require an entirely different study design that could not be implemented in the populations of horses we were using.

The distribution of body condition scores across populations of horses in Denmark is affected by the number of horses included in each subpopulation. However, the distributions of scores recorded for subpopulations represents a useful ratio of, for example, risk for being above or below ideal BCS and to identify protective factors for the given subpopulation.

The identification of patterns with regards to risk and protective factors is very useful in relation to individual management and population concerns regarding horse health and BCS.

## 5. Conclusions

The goals of this study were to learn more about the effect of use and training of horses on their health and condition using BCS as an indirect measure. Horses with above ideal BCS are 3.4 times more numerous than those with below ideal BCS, but both scenarios constitute a welfare problem. The patterns for the development of above and below ideal BCS differ and should be seen as separate problems. Being trained at moderate to high intensity levels provides a significant protective effect against being above ideal BCS. Below ideal BCS is not influenced by training. With regards to BCS, there are variations in the patterns of subpopulations attended by professions such as veterinarians, farriers, officials, and trainers.

## Figures and Tables

**Table 1:** Categories for level of training, including descriptions.

Category	Level or Intensity of Training	Description (Tick the Most Compatible Category)
1	No work	Maximum 1–2 times walked in hand/ridden per week
2	Low	Pleasure riding/hacking, rehabilitation, competes on rare occasions, low level, exercises 2–3 times per week for 30–60 min
3	Moderate	Riding lessons arena/outdoor/terrain, competes sometimes, exercises 3–5 times per week for 30–60 min
4	High	Training of high intensity, where the horse becomes hot/sweaty, competes regularly, exercises 4–6 times per week for 30–60 min
5	Very High	Training of very high intensity, sprinting, long duration, competing at elite level, exercises 5–7 times per week for 45–90 min

**Table 2:** Results of the bivariate analysis of the Body Condition Score and predictors. Kruskal–Wallis test, ordinal scale, and additional chi-square test, nominal scale (where data were sorted by <5 BCS (below ideal), 5–6 BCS (ideal), and >6 (above ideal)). A *p*-value < 0.05 indicates a significant difference between BCS and a parameter for each type of analysis.

	Kruskal–Wallis Ordinal Scale, *p*-Value	Chi-Square Nominal Scale, *p*-Value
Site of collection *	<0.0001	<0.0001
Sex	0.0188	0.0696
Age	<0.0001	<0.0001
Type	<0.0001	<0.0001
Discipline	<0.0001	<0.0001
Training level	<0.0001	<0.0001
Data collector’s profession	<0.0001	<0.0001 0.69 **
Data collector ***	<0.0001	<0.0001

* Site of collection is equal to type of consent. ** After adjustment for the site of collection, the effect of the profession of the data collector was not significant and it was excluded from the multivariate analysis. *** Data collector was included in the following multivariate analysis as a random effect.

**Table 3:** Odds ratio for multinominal logistic regression, ordinal scale. A *p*-value < 0.05 indicates significance between BCS and a parameter. For ORs, group ^a^ group ^b^ and group ^c^ are different. Odds ratio < 1 shows a decreased risk, odds ratio > 1 shows an increased risk.

Parameter		*Odds Ratio* Multinominal Logistic Regression Ordinal Scale
Site of collection	Private Yard	3.26 (*p = 0.0003*)
	Competition	1.00
Sex	Gelding	1.28 ^a^
	Mare	1.47 ^a^
	Stallion	1.00 ^a^
Age		1.06 (*p < 0.0001*)
Type	Cold-blood types	10.37 ^a^
	Icelandic horse	0.79 ^c^
	Race horse	0.20 ^c^
	Sport pony	1.18 ^c^
	Traditional pony	1.26 ^b^
	Mixed riding horses	1.00 ^c^
Discipline	None	1.31 ^a^
	Mixed	2.29 ^a^
	Hacking	2.49 ^a^
	Breeding	0.72 ^a^
	Riding school	1.98 ^a^
	Icelandic competition	3.18 ^a^
	Dressage	1.53 ^a^
	Showjumping	1.00 ^a^
Training level	+1 level	0.66 (*p < 0.0001*)
**Parameter**		***Odds Ratio*** **Multinominal Logistic Regression Ordinal Scale**
Site of collection	Private Yard	3.26 (*p = 0.0003*)
	Competition	1.00
Sex	Gelding	1.28 ^a^
	Mare	1.47 ^a^
	Stallion	1.00 ^a^
Age		1.06 (*p < 0.0001*)
Type	Cold-blood types	10.37 ^a^
	Icelandic horse	0.79 ^c^
	Race horse	0.20 ^c^
	Sport pony	1.18 ^c^
	Traditional pony	1.26 ^b^
	Mixed riding horses	1.00 ^c^

**Table 4:** Odds ratio for multinominal logistic regression, nominal scale. Data sorted by <5 BCS (below ideal), 5–6 BCS (ideal), and >6 (above ideal). A *p*-value < 0.05 indicates significance between BCS and a parameter. For OR’s, group ^a^ is different from group ^b^. Odds ratio < 1 shows a decreased risk, odds ratio > 1 shows an increased risk.

Parameter		*Odds Ratio* Multinominal Logistic Regression Nominal Scale
Site of collection	Private Yard above ideal BCS	1.00 ^b^
	Private Yard below ideal BCS	1.00 ^b^
	Competition above ideal BCS	0.13 (*p < 0.01*)
	Competition below ideal BCS	3.02 ^b^
Age	Age above ideal BCS	1.05 ^b^
	Age below ideal BCS	1.06 ^b^
Type	Cold-blood type above ideal BCS	16.78 ^a^
	Cold-blood type below ideal BCS	0.02 ^b^
	Icelandic horse above ideal BCS	0.83 ^b^
	Icelandic horse below ideal BCS	0.45 ^b^
	Racehorse above ideal BCS	0.38 ^b^
	Racehorse below ideal BCS	13.66 ^b^
	Sport pony above ideal BCS	1.48 ^b^
	Sport pony below ideal BCS	0.82 ^b^
	Trad. pony above ideal BCS	3.27 ^b^
	Trad. pony below ideal BCS	0.48 ^b^
	Mixed riding horse above ideal BCS	1.00 ^b^
	Mixed riding horse below ideal BCS	1.00 ^b^
Discipline	None above ideal BCS	1.07 ^b^
	None below ideal BCS	6.40 ^b^
	Mixed above ideal BCS	2.31 ^b^
	Mixed below ideal BCS	0.00 ^b^
	Hacking above ideal BCS	2.85 ^b^
	Hacking below ideal BCS	3.94 ^b^
	Breeding above ideal BCS	0.62 ^b^
	Breeding below ideal BCS	3.69 ^b^
	Riding school above ideal BCS	1.24 ^b^
	Riding school below ideal BCS	0.02 ^b^
	Icelandic comp. above ideal BCS	4.66 ^b^
	Icelandic comp. below ideal BCS	14.12 ^b^
	Dressage above ideal BCS	2.57 ^b^
	Dressage below ideal BCS	0.35 ^b^
	Showjumping above ideal BCS	1.00 ^b^
	Showjumping below ideal BCS	1.00 ^b^
Training level	+1 level increase in BCS	0.46 (*p = 0.02*)
	+1 level decrease in BCS	0.94 ^b^

**Table 5:** Results of the multivariate analysis of the Body Condition Score and predictors. Multinominal logistic regression analysis, ordinal scale, and the additional multinominal logistic regression analysis, nominal scale, where data were sorted by <5 BCS (below ideal), 5–6 BCS (ideal), and >6 (above ideal). A *p*-value < 0.05 indicates significance between BCS and a parameter for each type of analysis.

Parameter	Multinominal Logistic Regression Ordinal Scale, *p*-Value	Multinominal Logistic Regression Nominal Scale, *p*-Value
Site of collection *	0.0056	0.0118
Sex **	0.4554	-
Age	0.0030	0.0858
Type	0.0643	0.0504
Discipline	0.3093	0.4938
Training level	0.0093	0.0460

* Site of collection is equal to type of consent. Site of collection had a close relationship with profession. ** Sex was excluded from additional analysis due to the lack of correlation in the bivariate chi-square test for the nominal scale.

**Table 6:** Distribution of below ideal, ideal, and above ideal BCS in relation to the type of horse.

Type of Horse	<5 BCS % below Ideal	5–6 BCS % Ideal	>6 BCS % above Ideal	N (1118)
Cold-blooded	0.00	20.7	79.3	29
Icelandic	3.4	63.0	33.6	146
Race	28.6	57.1	14.3	7
Sport pony	5.0	83.0	12.0	300
Traditional pony	3.6	50.0	46.4	28
Riding horse	5.1	84.7	10.2	608

**Table 7:** Distribution of below ideal, ideal, and above ideal BCS for horses in relation to age.

Age/Year	<5 BCS % below Ideal	5–6 BCS % Ideal	>6 BCS % above Ideal	N (1118)
≤5	5.2	85.2	9.6	135
6–10	3.3	85.4	11.3	460
11–15	6.1	74.9	19.0	326
16–20	3.9	70.0	26.2	130
21–25	7.1	58.9	33.9	56
26–30	27.3	36.4	36.4	11

**Table 8:** Distribution of below ideal, ideal, and above ideal BCS for horses in relation to the training level.

Training Level	<5 BCS % below Ideal	5–6 BCS % Ideal	>6 BCS % above Ideal	N (1118)
**1. No training**	6.9	54.6	38.5	130
**2. Low**	4.8	51.2	44.0	166
**3. Moderate**	4.2	86.9	8.9	595
**4. High**	5.0	91.4	3.6	222
**5. Very High**	20.0	80.0	0.0	5

**Figure A1:**
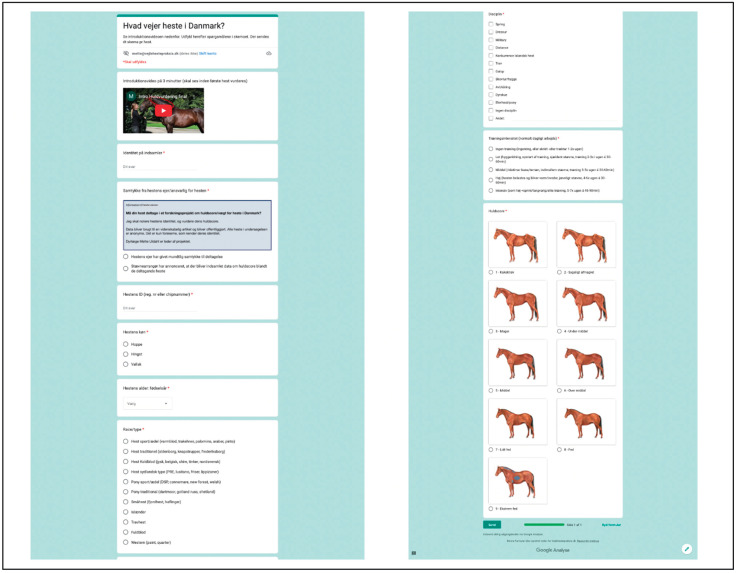
PDF copy of the survey questionnaire.

## Data Availability

All data are available from the first author.

## Appendix B

**Link B1** instruction video: https://youtu.be/u_24zySuQp4 (accessed on 19 December 2022).
